# Dysglycemia and Arthroplasty Outcomes: A Review

**DOI:** 10.7759/cureus.10239

**Published:** 2020-09-04

**Authors:** Talal Alkindy

**Affiliations:** 1 Orthopaedics, University of Tabuk, College of Medicine, Tabuk, SAU

**Keywords:** the impact of diabetes mellitus and prediabetes on arthroplasty outcomes, a review

## Abstract

Arthroplasty (ART) is a common surgery and it is on the rise worldwide due to increasing longevity and osteoarthritis. The effects of perioperative hyperglycemia on the outcomes are largely unknown and the current review aimed to assess the impact of perioperative hyperglycemia on ART outcomes.

The literature in PubMed and Google Scholar was searched for relevant articles published in the last ten years up to February 2020. The keywords knee ART, hip ART, diabetes mellitus (DM) impaired fasting glucose (IFG), impaired glucose tolerance (IGT), and impaired glycated hemoglobin (HbA1c)were used. Among the 113 articles retrieved, 34 full-texts were eligible, and only 21 studies (17 from the USA, three from Europe, and two from Asia) met the inclusion criteria for the systematic review. The authors' names, year of publication, country, type of study, number of patients, and duration of the study were reported.

The studies reviewed showed high ART complication rates including infections, loosening, increasing severity and depth of infection, more pain, and higher costs with high perioperative hyperglycemia. The cut-off glycated hemoglobin values associated with complications ranged from 6.7 to >8.

## Introduction and background

Arthroplasty (ART) is not uncommon. Seven million people were living with knee and hip ART in the US in the year 2010 with a prevalence of 0.83% to 10.38% depending on age and sex, and the procedure is on the rise worldwide due to the burden of osteoarthritis and increasing longevity [1,2]. ART, like other surgery, is not without local and general complications including loosening, hemorrhage, and deep vein thrombosis [3]. Currently, 374 million people 20-79 years old have impaired glucose tolerance (IGT; prediabetes) and 463 are suffering from diabetes mellitus (DM) [4], most of whom are undiagnosed. The impact of DM and prediabetes on ART is a matter of controversy as is which test (impaired fasting glucose (IFG), IGT, or glycated hemoglobin) is preferred. Thus, we conducted this review to assess the impact of DM and prediabetes on ART.

## Review

Methods

Eligibility Criteria According to PICOS

Type of study: All articles in English language (observational studies) assessing the effects of diabetes and prediabetes on ART (both upper and lower limb ART) outcomes published during the last ten years up to February 2020 were eligible. Studies were eligible if they were longitudinal (prospective or retrospective studies, prospective cohort, case-control, nested case-control that reported the association of ART (knee or shoulder) outcomes in term of superficial or deep infections, loosening, cost, hospital stay, and pain with dysglycemia (impaired fasting plasma glucose, IGT, or high HbA1c). Studies examining any type of infection were approached (periprosthetic joint infection, deep infection, deep surgical site infection, or deep prosthetic infection). We complemented the search by manual scanning of reference lists of identified articles. The search was limited to studies conducted in humans.

Exclusion criteria: Case series studied, case reports, and animal studies and studies published in languages other than English were excluded. Operations during childhood were excluded from the study.

Type of participants: Adult patients who underwent ART (in the upper and lower limbs) and were diagnosed with diabetes or prediabetes during the last ten years up to February 2020.

Type of outcome measures: We included studies if they measured at least one of the following outcome measures: infections (deep or superficial), wound complications, postoperative pain, aseptic loosening, length of stay, hospital costs, and mortality. 

Information sources and search methods: A systematic manual search was conducted in PubMed (including ahead of print and Epub) and the first hundred articles of Google Scholar databases published in the last ten years up to February 2020. The keywords used were those related to exposure including knee ART, hip ART, DM-impaired fasting glucose, impaired glucose tolerance, dysglycemia, undiagnosed diabetes, and impaired HbA1c with those related to outcomes (e.g., wound infection, loosening, pain, cost, and hospital stay with protean AND or OR. Among the 113 articles retrieved, 34 full-texts were eligible, and only 22 studies (17 retrospective studies, two prospective cohorts, two case-control studies, and one cross-sectional study) met the inclusion criteria for the systematic review. The author's name, year of publication, country, type of study, number of patients, and the duration of the study were reported.

**Figure 1:**
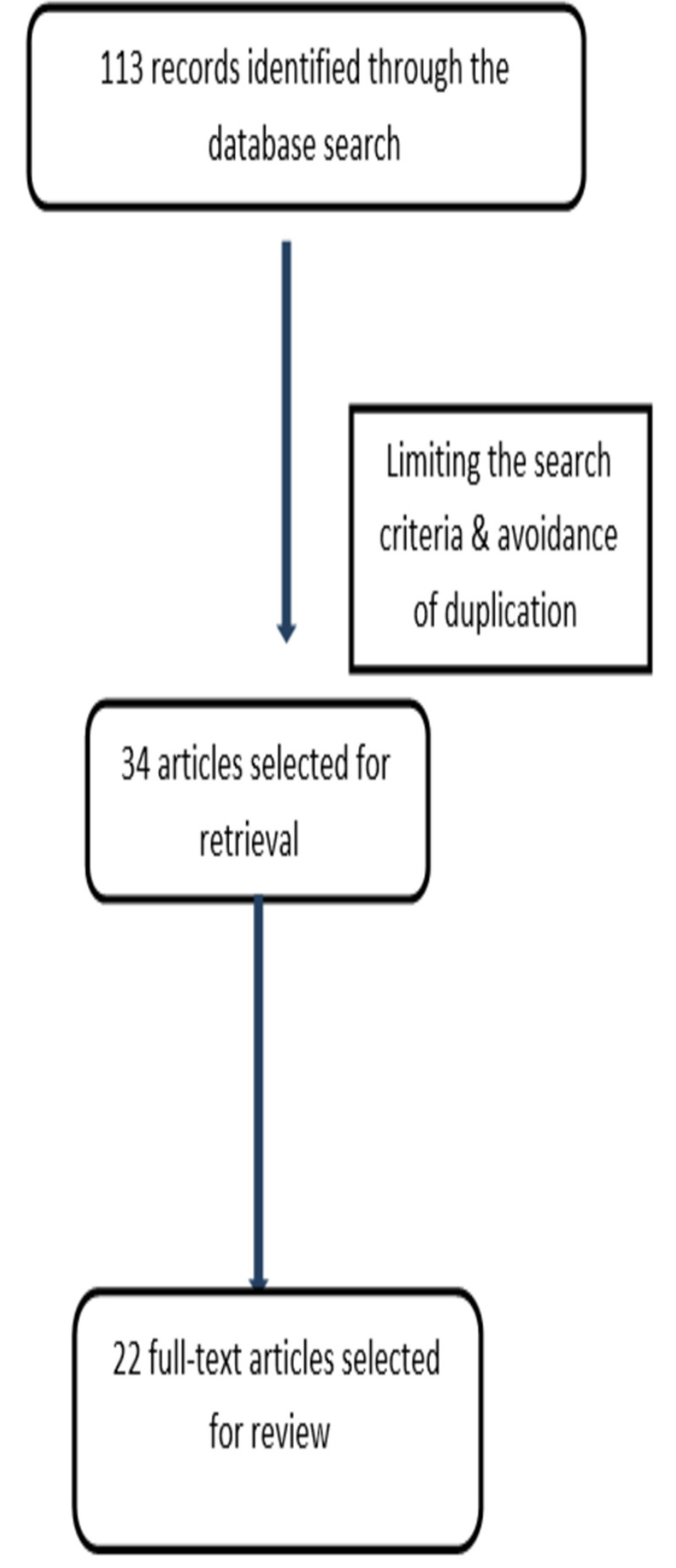
The different phases of the literature search

Titles and abstracts were screened by the author and full texts retrieved for the manuscripts found relevant for the topic and excluded clearly irrelevant articles. Additional articles were searched and identified through hand searching of the bibliography. The retrieved full-text articles were assessed for eligibility for inclusion and data were extracted by the authors using proforma. Any disagreement in the selection of articles and data was solved by consensus. Figure 1 illustrated the different phases of the literature search.

Assessment of Risk of Bias in Included Studies

For the assessment of study quality, the risk of bias assessment according to Newcastle-Ottawa was applied in Table 1.

**Table 1:** Quality of the selected studies by using the Newcastle-Ottawa scale

Author	Selection	Compatibility and outcome	Total score
Mravoic et al. [5]	4	3	7
Jamsen et al. [6]	4	5	9
Strykler et al. [7]	4	5	9
Adams et al. [8]	4	5	9
Chastil et al. [9]	4	3	7
Maradit Kremers et al. [10]	4	3	7
Rajamäki et al. [11]	4	4	8
Hwang et al. [12]	4	4	8
Brock et al. [13]	4	4	8
Maradit Kremers et al. [14]	4	5	9
Cancienne et al. [15]	4	3	7
Tarabichi et al. [16]	4	4	8
Capozzi et al. [17]	4	3	7
Lavernia et al. [18]	4	3	7
Cancienne et al. [19]	4	3	7
Godshaw et al. [20]	4	3	7
Kurowicki et al. [21]	4	4	8
Kheir et al. [22]	4	4	8
Shohat et al. [23]	4	3	7
Lenguerrand et al. [24]	4	4	8
Ryan et al. [25]	4	3	7
Shohat et al. [26]	4	4	8

Results

A total of 113 studies were identified through the database search. Out of the 34 full texts retrieved, only 22 were included in the review (17 retrospective studies, two prospective cohorts, two case-control studies, and one cross-sectional), 17 were published in the USA, three from Europe, and two were from Asia, 104,342 patients included. All the retrospective studies showed the association of perioperative hyperglycemia with ART infection and loosing; however, there are mixing results regarding the HbA1c cut-off at which infections and other complications were observed (the HbA1c ranged from 6.7 to 8), the two prospective cohorts concluded the increased complications among both diagnosed and undiagnosed patients with diabetes and suggested that fructosamine is more accurate than the HbA1c (293 µmol/l was identified as the optimal cut-off associated with complications). The case-control studies retrieved showed mixed results with one study stated that preoperative glycemic control on the length of stay, hospital costs, or rate of short-term postoperative complications, while the other concluded less improvement observed among patients with DM at I year and more at HbA1c > 8. The results showed that both postoperative hyperglycemia and the HbA1c were shown to be associated with joint infections; however, this effect was lost after controlling for BMI, type of surgery, and operative time. The studies showed conflicting results regarding the length of stay, hospital costs, or rate of short-term postoperative complications. The association was again attenuated after controlling for obesity and other comorbidities. Table 2 depicted the association between ART outcomes, diabetes, and prediabetes.

**Table 2:** The relationship between glycemia and diabetic ART ART: arthroplasty, DM: diabetes mellitus, HbA1c: glycated hemoglobin, RBS: random blood sugar, FBS: fasting blood sugar.

Author	Year	Country	Study type	Patients	Result
Mraovic et al. [5]	2011	USA	Retrospective	1948	DM and morning postoperative hyperglycemia were predictors for postoperative infection, also, high BMI and comorbidities
Jämsen et al. [6]	2012	Finland	Retrospective	7181 total knee and hip ART	DM doubled the rate of infections especially when associated with morbid obesity, postoperative of 124 mg is suggested.
Stryker et al. [7]	2013	USA	Retrospective	1702 joint ART	Postoperative blood glucose of >200 mg/dL or a preoperative hemoglobin A1C level of >6.7% are predictors for wound complications
Adams et al. [8]	2013	USA	Retrospective	40,491 total knee ART	No differences between diabetes HbA1c < 7 or more and comparators regarding complications
Chrastil et al. [9]	2015	USA	Retrospective	13,272 joint ART	Increased infections with perioperative hyperglycemia, but not HbA1c (high mortality)
Maradit Kremers et al. [10]	2015	USA	Retrospective	20,171 total hip and knee ART	No association of perioperative hyperglycemia and diabetes with prosthetic joint infections after controlling for BMI, type of surgery, and operative time.
Rajamäki et al. [11]	2015	Finland	Cross-sectional	193 knee and hip	Increased postoperative pain among patients with diabetes
Hwang et al. [12]	2015	Korea	Retrospective	462 total knee ART	FBS ≥200 mg/dl and HbA1c ≥8 strongly correlate with superficial infections
Brock et al. [13]	2017	UK	Case-control	200 total knee ART	Less improvement observed among patients with DM at I year and more at HbA1c > 8
Maradit Kremers et al. [14]	2017	USA	Retrospective	16,085 knee and hip ART	High preoperative hyperglycemia is a potential risk factor for aseptic loosening
Cancienne et al. [15]	2017	USA	Retrospective	7763 total hip ART	HbA1c is a poor predictor of deep infections, however, a level ≥7.5 increased infections
Tarabichi et al. [16]	2017	USA	Retrospective	1645 knee and hip ART	HbA1c 7.7% is more indicative of infection
Capozzi et al. [17]	2017	USA	Retrospective	663 knee and hip ART	Prediabetes in 31% and diabetes in 2.6%
Lavernia et al. [18]	2017	USA	Case-control	120 total joint ART followed for 5.9 years	No effects of preoperative glycemic control on the length of stay, hospital costs, or rate of short-term postoperative complications.
Cancienne et al. [19]	2018	USA	Retrospective	Shoulder ART	Higher infection at perioperative HbA1c > 8
Godshaw et al. [20]	2018	USA	Retrospective	773 total joint ART	HbA1c of 7.45 is suggested (correlated with RBS of 200 mg/dl postoperatively)
Kurowicki et al. [21]	2018	USA	Retrospective	Total hip ART	Higher perioperative HbA1c levels increase the cost
Kheir et al. [22]	2018	USA	Retrospective	24,857 primary total joint ART	Even mild hyperglycemia was significantly associated with periprosthetic joint infection. Postoperative cut-off was 137 mg/dl.
Shohat et al. [23]	2018	Israel, USA	Prospective cohort	1461 knee ART	Complications noted, no differences between diagnosed and undiagnosed DM.
Lenguerrand et al. [24]	2018	UK	Retrospective	Hip and knee ART (587)	Hospital stay, pain, and complications increased and attenuated after controlling for obesity and comorbidities.
Ryan et al. [25]	2019	USA	Retrospective	1406	Perioperative blood glucose and HbA1c are not predictive of postoperative infections; however, deep and prolonged infections were observed.
Shohat et al. [26]	2019	USA	Prospective cohort	1119	Fructosamine level of 293 µmol/l was identified as the optimal cut-off associated with complications.

Discussion

In the current review, a retrospective study conducted in the USA [5] and including 1948 patients found that DM and morning perioperative hyperglycemia were predictors for postoperative infection of lower limb ART; the findings were supported by Jämsen et al. [6] from Finland and Stryker et al. [7] in the USA. The latter study also found an HbA1c of 6.7 is a predictor of postoperative wound infection, and in contradiction, Adams et al. [8] found no differences between patients with diabetes and counterparts without the disease regarding thrombosis, myocardial infarction, and readmission. A cut-off of ≥7 of the HbA1c was found to be associated with mortality among patients who underwent total ART. However, there was no association with perioperative joint infection [9]. The findings of Maradit Kremers et al. [10] related postoperative infection to high body mass index, type of surgery, and length of operative time; the findings of increased pain after ART among patients with diabetes could be due to diabetic neuropathy [11]. In the current review, an FBS of >200 mg and an HbA1c of ≥8 were associated with superficial infections and slow improvement [12,13]. Maradit Kremers and colleagues in their more recent study [14] found that high preoperative hyperglycemia is a potential risk factor for aseptic loosening, while two retrospective studies with large samples [15,16] showed the association of deep infections with A HbA1c of 7.5 and 7.7. Capozzi et al. [17] reported that nearly one-third of patients who underwent ART were prediabetes, and a case-control study published in the USA [18] showed no effects of preoperative glycemic control on length of stay, hospital costs, or rate of short-term postoperative complications. However, the study conclusion was based on the glycated hemoglobin and only 120 patients were included. Further studies showed conflicting cut-off regarding the association of the glycated hemoglobin and perioperative infections, 8 and 7.45, respectively [19,20]. Another study [21] reported the association of higher glycated hemoglobin and pain, while Kheir et al. [22] in their retrospective study showed that even mild hyperglycemia was significantly associated with periprosthetic joint infection. The postoperative cut-off was 137 mg/dl, and Sohat et al. [23] found no difference between known cases of DM and those who were not diagnosed in terms of postoperative infections. Further studies based their conclusion on the glycated hemoglobin [24] and stated that hospital stay, pain, and complications increased and attenuated after controlling for obesity and comorbidities. A recent study published in the USA [25] found that perioperative blood glucose and HbA1c are not predictive of postoperative infections; however, deep and prolonged infections were observed, and Shohat et al. [26] found that a fructosamine level of 293 µmol/l was identified as the optimal cut-off associated with complications.

## Conclusions

High perioperative hyperglycemia was associated with more ART complications. However, the complications were attenuated after controlling for obesity and other comorbidities. Controlling high body mass index and comorbidities may be needed before ART among patients with diabetes and prediabetes.
